# Supplementary material for: Extracellular matrix stiffness and cell contractility control RNA localization to promote cell migration
Source: Nat Commun. 2017 Oct 12;8:896. doi: 10.1038/s41467-017-00884-y (PMC5638855; doi:10.1038/s41467-017-00884-y)
Supplement: Supplementary file 1 — Supplementary Information [file 41467_2017_884_MOESM1_ESM.pdf]

Supplementary Figure 1

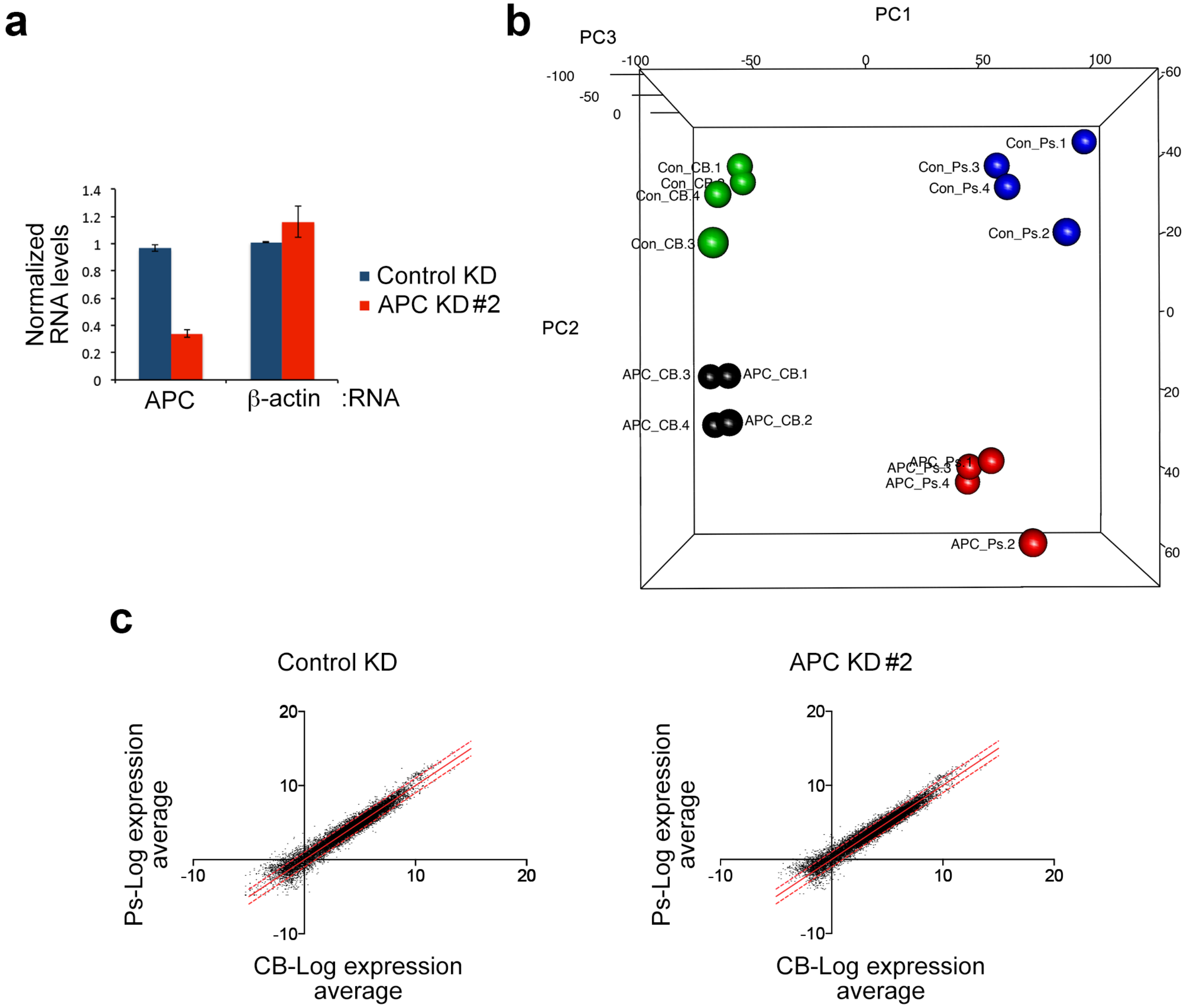

Supplementary Figure 1: RNA levels in control and APC knockdown cells and scatter plots of RNA-Seq data. (**a**) Total RNA was analyzed by RT-qPCR to detect APC or β-actin mRNAs. Levels were normalized to GAPDH mRNA. N=4. Error bars: standard error. (**b**) 3D principal component (PC) scatter plots. Each of 4 replicate protrusion (Ps) and cell body (CB) samples from control (Con) or APC knockdown cells, analyzed by RNA-Seq, were plotted on a 3d PCA scatter plot. (**c**) Scatter plots of log-expression values of all RNAs detected by RNA-Seq analysis, in Ps and CB samples. Values are averaged from 4 independent biological replicates. Dotted red lines mark fold change>2 cutoffs towards Ps or CB.

Supplementary Figure 2

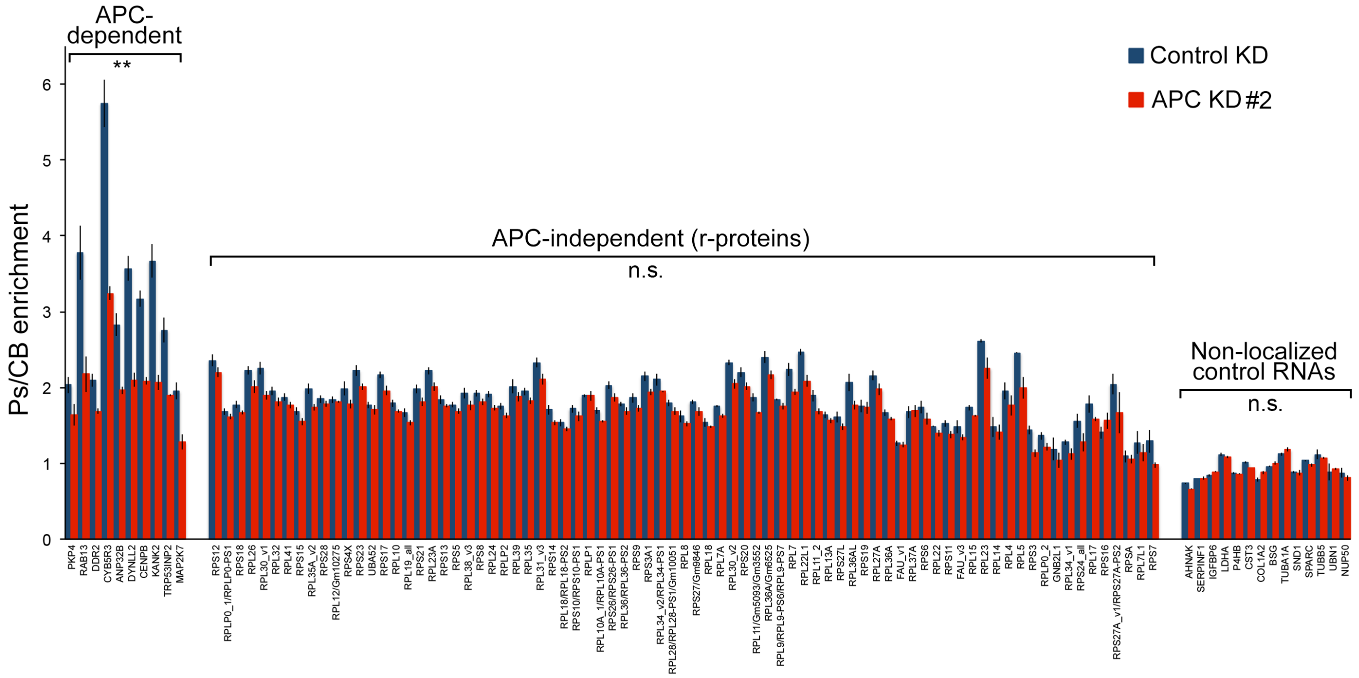

Supplementary Figure 2: Ps/CB enrichment ratios from control or APC knockdown cells (APC KD#2) detected through nanoString analysis. The indicated RNAs were detected through nanoString analysis of protrusion (Ps) and cell body (CB) fractions, and Ps/CB enrichment ratios were calculated (n=3; error bars: standard error). Changes in enrichment ratios between control and APC knockdown samples are significant for all APC-dependent RNAs (**: $p<0.0001$ by 2-way analysis of variance with Bonferroni's multiple comparisons test, except for Ddr2 ($p=0.035$) and Pkp4 ($p=0.051$)). Changes of APC-independent and control RNAs are non-significant (n.s.).

Supplementary Figure 3

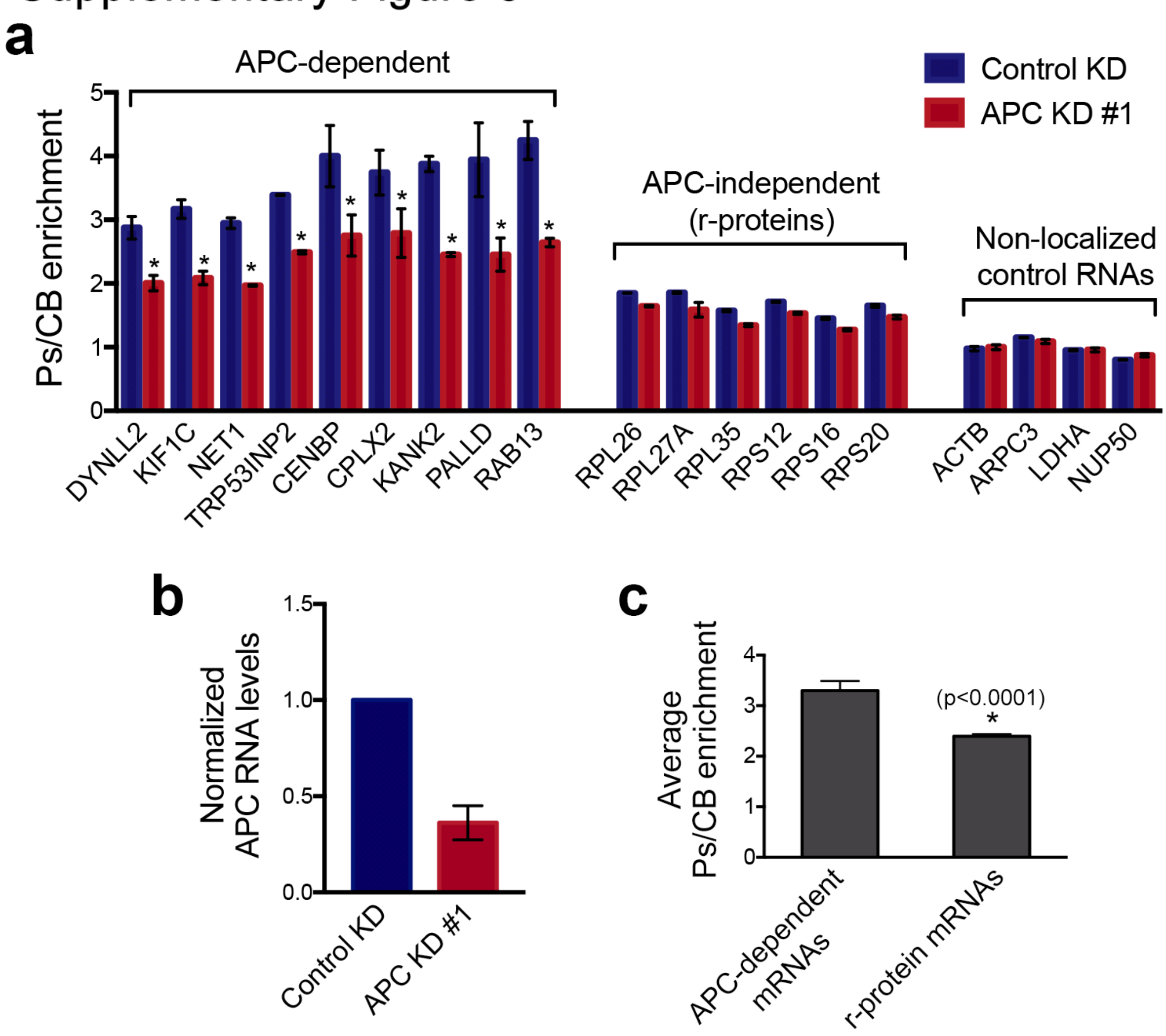

Supplementary Figure 3: Ps/CB enrichment ratios from control or APC knockdown cells (APC KD#1) detected through nanoString analysis. (**a**) Control or APC knockdown cells (KD #1) were fractionated into Ps and cell body (CB) fractions. The indicated RNAs were detected through nanoString analysis to calculate Ps/CB enrichment ratios (n=3; error bars: standard error). *: p-value<0.03, by analysis of variance with Bonferroni's multiple comparisons test against the corresponding control. (**b**) Normalized APC RNA levels in control and APC knockdown cells (KD #1) detected by RT-qPCR. N=5. Error bars: standard error. (**c**) Average Ps/CB enrichment ratios of all APC-dependent RNAs and r-protein mRNAs detected by RNA-Seq analysis.

Supplementary Figure 4

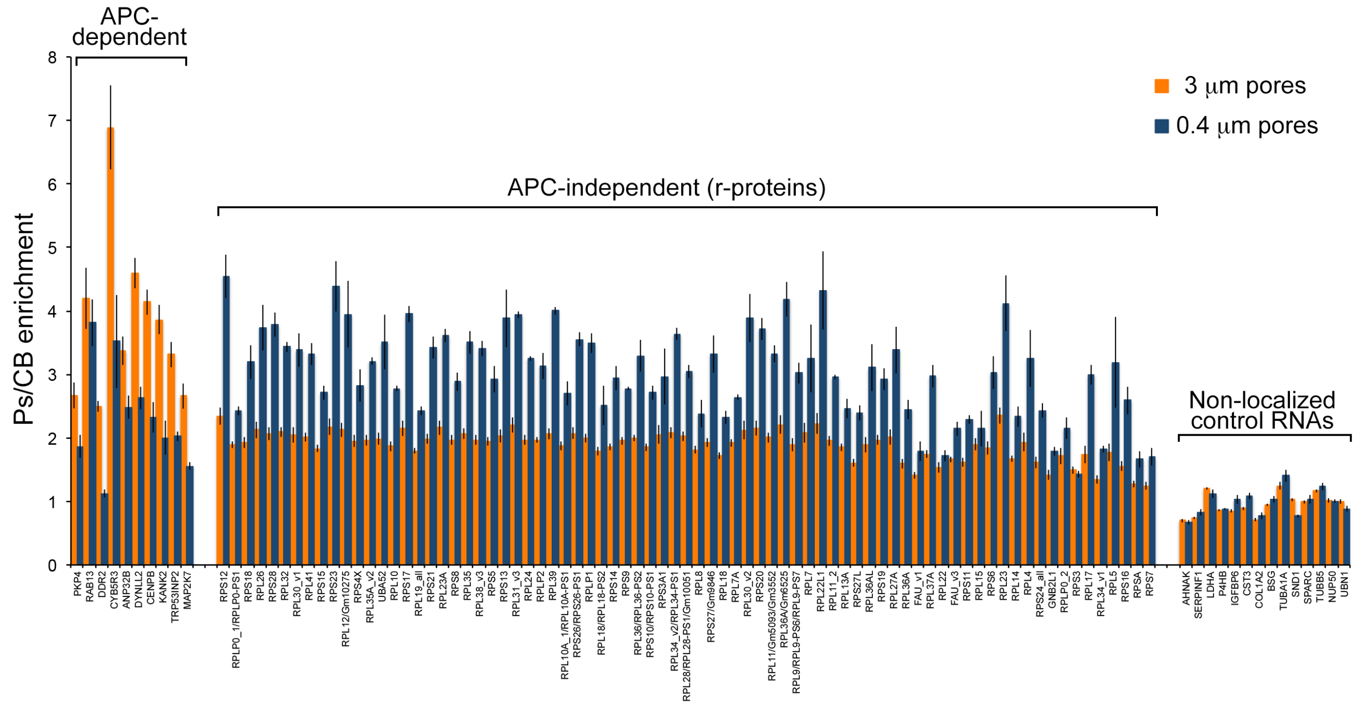

Supplementary Figure 4: Ps/CB enrichment ratios from cells extending protrusions through 3μm or 0.4μm pores. The indicated RNAs were detected through nanoString analysis of protrusion (Ps) and cell body (CB) fractions, and Ps/CB enrichment ratios were calculated (n=3; error bars: standard error).

Supplementary Figure 5

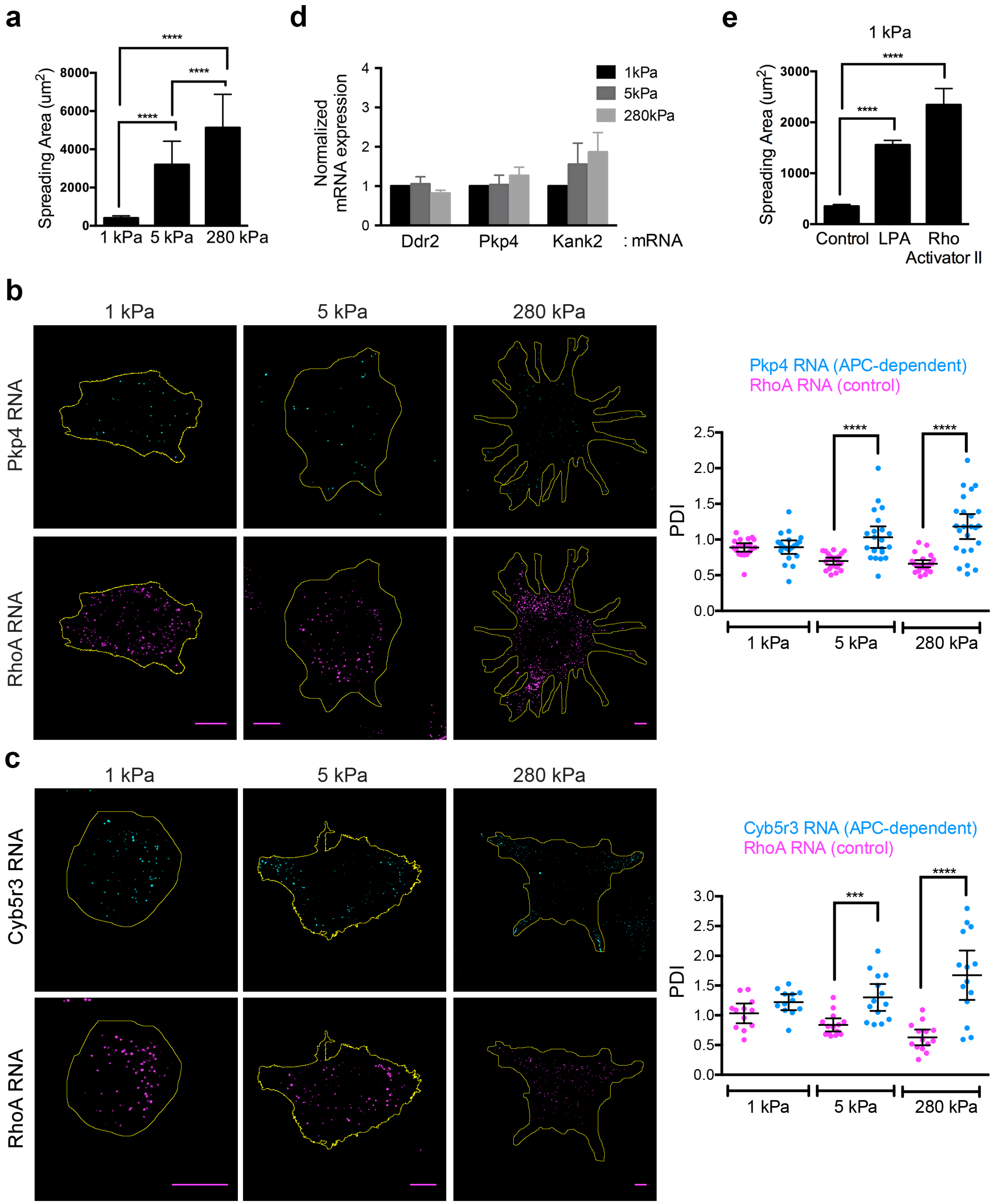

Supplementary Figure 5: Effect of substrate stiffness on cell spreading area, RNA levels and peripheral localization. (**a**) Average spreading area of cells plated on substrates of the indicated

stiffness. N values: 25, 33 and 23 cells respectively. Error bars: SD. (**b**-**c**) Distributions of APC-dependent and control RNAs in response to increasing stiffness. Representative in situ hybridization images showing the distribution of the *Pkp4* (APC-dependent) and *RhoA* (control) RNA (**b**), or the *Cyb5r3* (APC-dependent) and *RhoA* (control) RNA (**c**), and corresponding PDI values from a population of cells. For PDI quantifications, bars represent the mean with 95% confidence interval. Points indicate individual cells analyzed in at least two or more independent experiments. Scale bars: 10μm. (**d**) Expression levels of APC-dependent RNAs in response to increasing stiffness. Cells were plated on 1, 5 or 280 kPa substrates and total RNA was analyzed by droplet digital PCR. Expression levels of the APC-dependent RNAs *Ddr2*, *Pkp4* and *Kank2* were normalized to *GAPDH* and *actin* RNA levels and plotted relative to the 1 kPa samples. N=3, error bars: SEM. (**e**) Changes in average spreading area of cells plated on 1 kPa substrates upon treatment with LPA or Rho Activator II. N values: 31, 28 and 28 cells respectively. Error bars: SD. P-values: ****<0.0001, ***<0.001 by analysis of variance with Bonferroni's multiple comparisons test (a, e) or Mann Whitney test (b, c).

Supplementary Figure 6

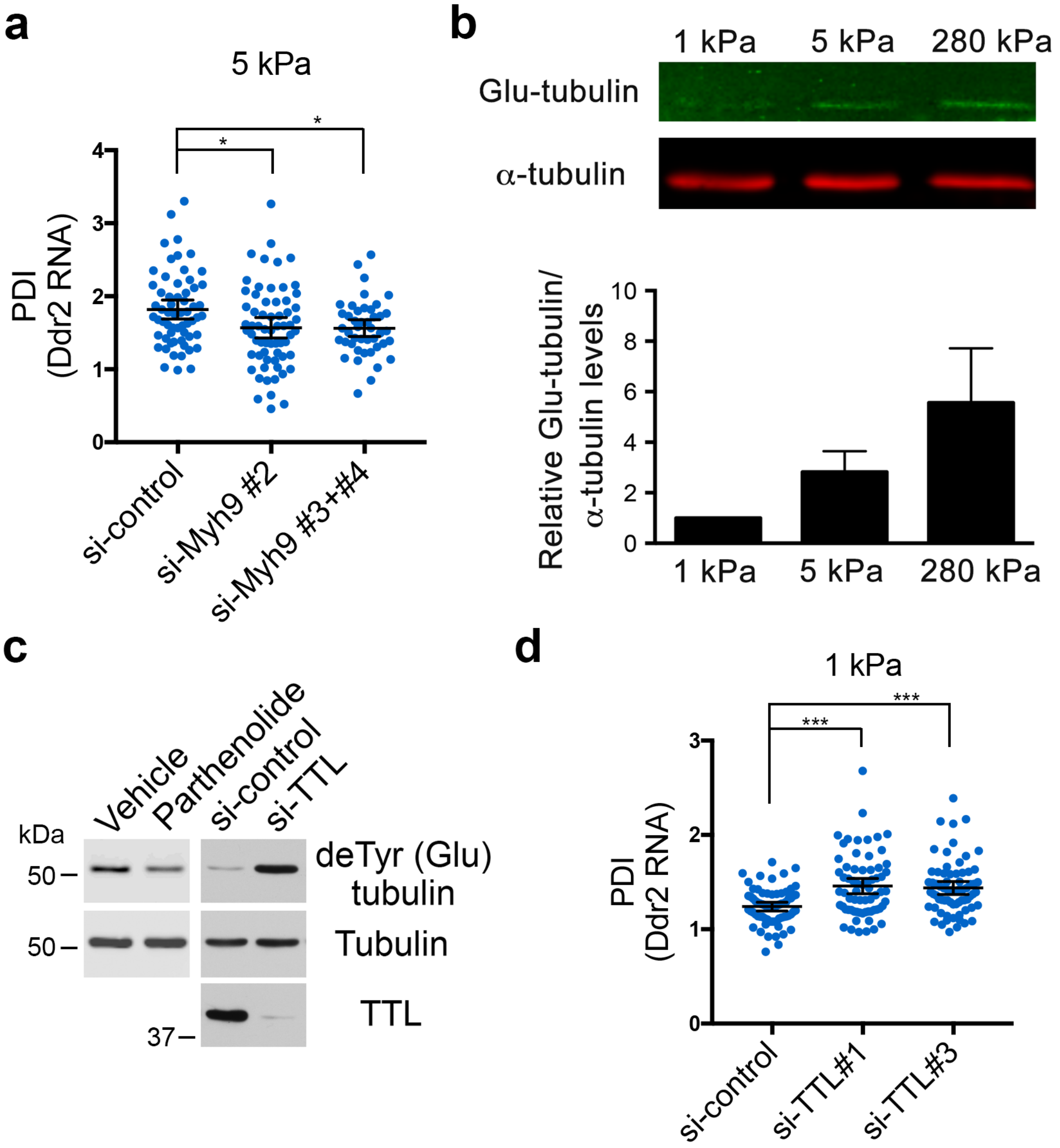

Supplementary Figure 6: Effect of myosin and detyrosinated microtubule levels on peripheral RNA localization. (**a**) PDI values of Ddr2 RNA from cells transfected with control si-RNAs or the indicated siRNAs against myosin heavy chain IIA, Myh9. Cells were plated on 5 kPa substrates. (**b**) Glu-tubulin levels on substrates of different stiffness. Cells grown on 1, 5 or 280 kPa substrates were analyzed by Western blot to detect Glu-tubulin or total α-tubulin levels. Normalized Glu-tubulin levels from two independent experiments are plotted. Error bars: SD. (**c**) Changes in Glu-tubulin levels upon treatment with parthenolide or TTL knockdown. NIH/3T3 cells were treated with parthenolide, or were transfected with control si-RNAs or si-RNAs against TTL. Cell extracts were analyzed by Western blot to detect the indicated proteins. (**d**) PDI values of Ddr2 RNA from cells transfected with control si-RNAs or

the indicated siRNAs against TTL. Cells were plated on 1 kPa substrates. For PDI quantifications, bars represent the mean with 95% confidence interval. Points indicate individual cells analyzed in at three or more independent experiments. P-values: ***<0.001, *<0.05 by analysis of variance with Bonferroni's multiple comparisons test.

Supplementary Figure 7

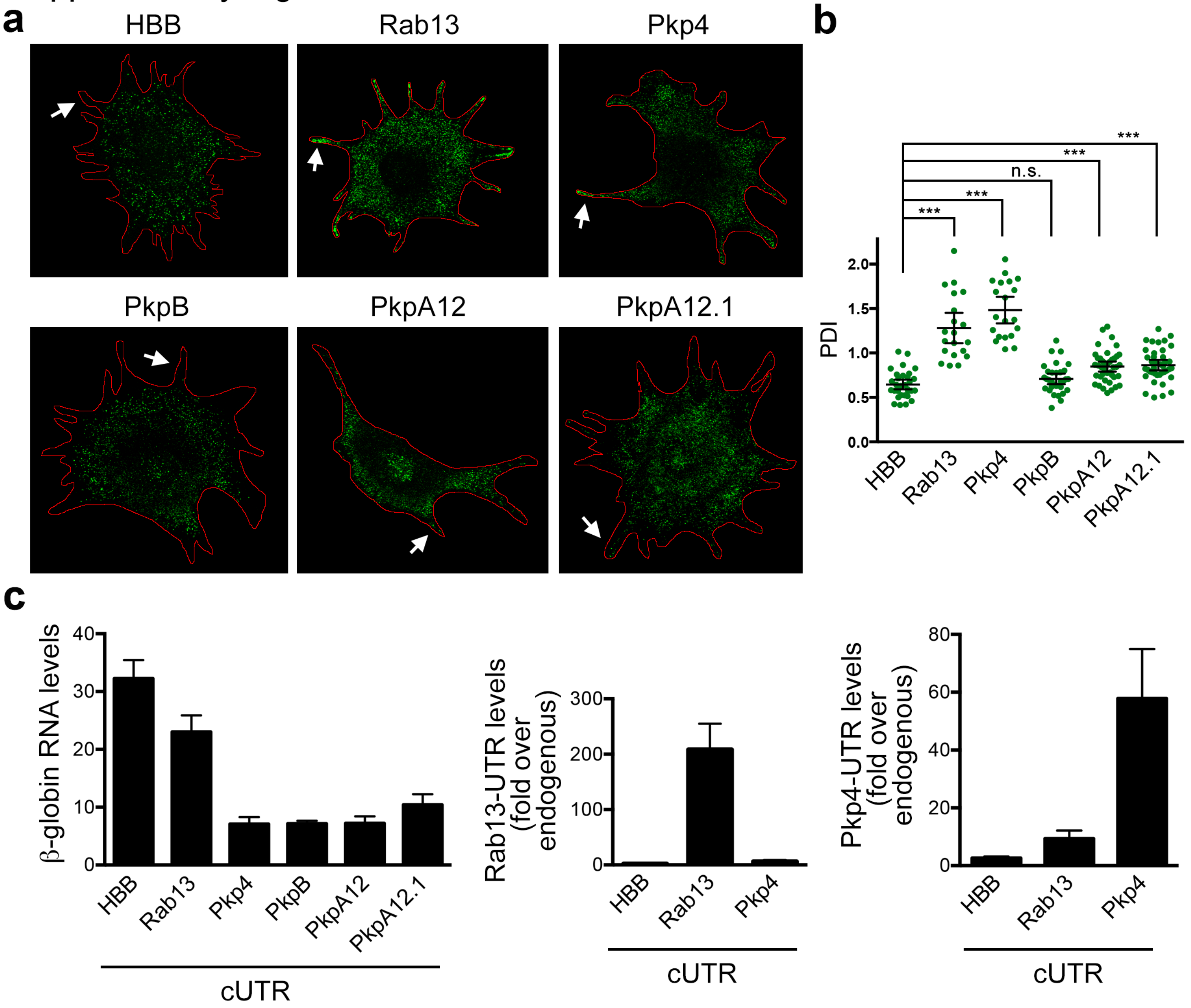

Supplementary Figure 7: Characterization of cell lines expressing cUTR constructs. (**a**) Distributions of exogenously expressed β-globin RNAs carrying the indicated 3'UTRs. Cell lines, stably expressing β-globin constructs with the indicated UTRs were analyzed by in situ hybridization to detect the β-globin RNA. Arrows point to protrusions enlarged in Fig. 7a. (**b**) PDI values of β-globin RNA from cells shown in (a). Bars represent the mean with 95% confidence interval. ***: p-value<0.001 by analysis of variance with Bonferroni's multiple comparisons test. (**c**) Relative expression levels of exogenously expressed β-globin RNAs carrying the indicated 3'UTRs. RT-PCR analysis of total RNA from cell lines expressing β-globin constructs with the indicated UTRs. Left panel: β-globin RNA levels. Middle and right panels: Rab13 or Pkp4 UTR levels, respectively, to assess degree of overexpression relative to the

corresponding endogenous transcripts. All levels were normalized to GAPDH RNA levels and were expressed relative to the uninduced HBB control (N=5-6; error bars: standard error).

Supplementary Figure 8

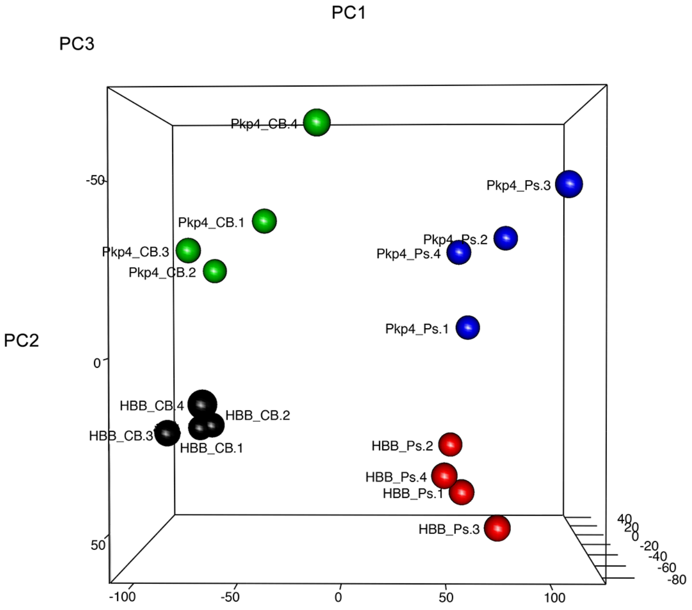

Supplementary Figure 8: 3D principal component (PC) scatter plots of RNA-Seq data. Each of 4 replicate protrusion (Ps) and cell body (CB) samples from control (HBB) or Pkp4 cUTR expressing cells (Pkp4), analyzed by RNA-Seq, were plotted on a 3d PCA scatter plot.

Supplementary Figure 9

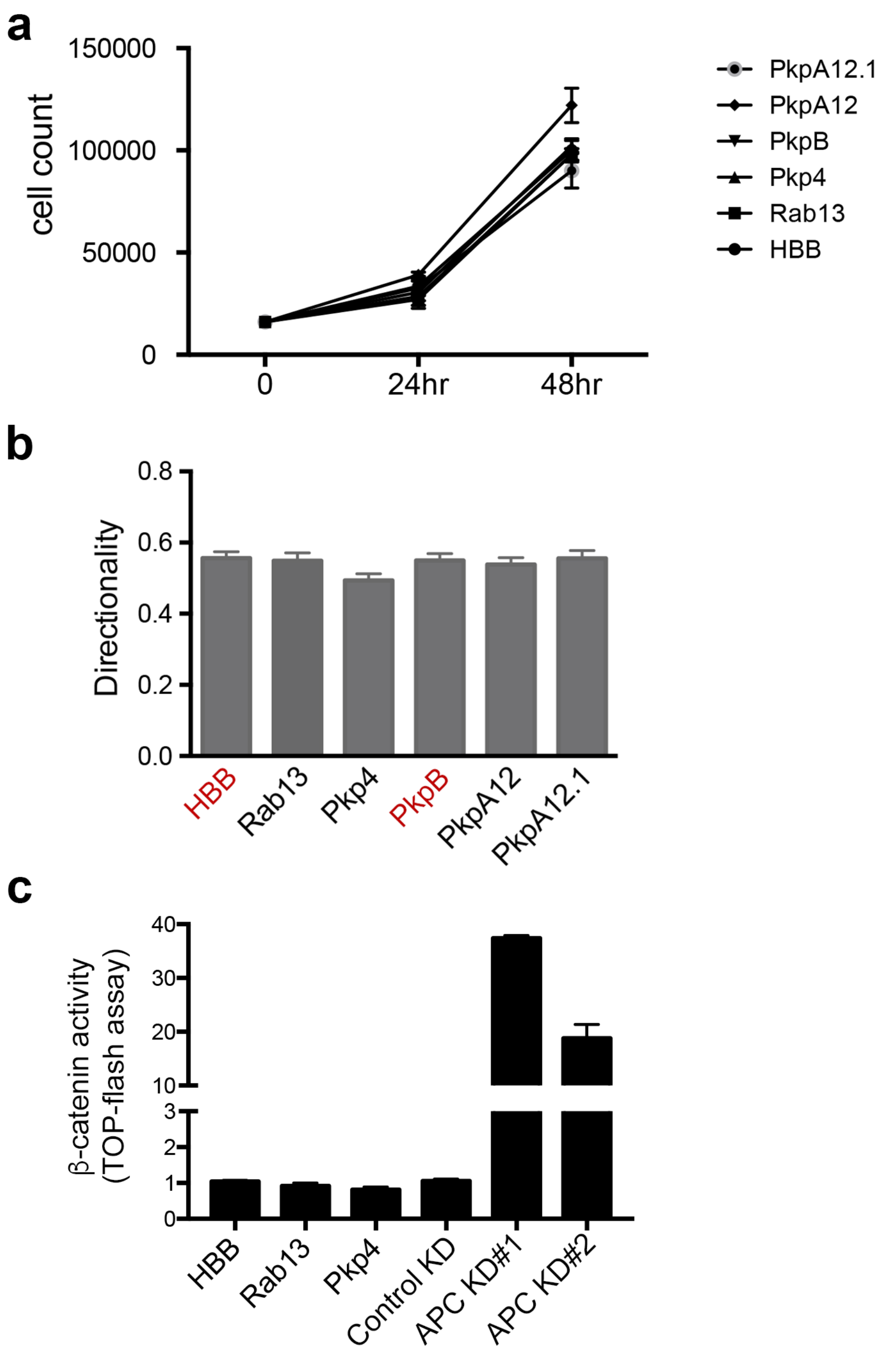

Supplementary Figure 9: Effect of cUTR expression on cell proliferation, directionality and β-catenin transcriptional activity. (**a**) Growth curves of cell lines stably expressing β-globin constructs with the indicated UTRs. (**b**) Average directionality values of randomly migrating cells. Values were derived from multiple individual cell tracks in two independent experiments observing 40-70 cells in each. (**c**) β-catenin transcriptional activity of the indicated cell lines was measured using the TOP-flash assay. Normalized luciferase levels from 3 independent experiments are reported.

Supplementary Figure 10

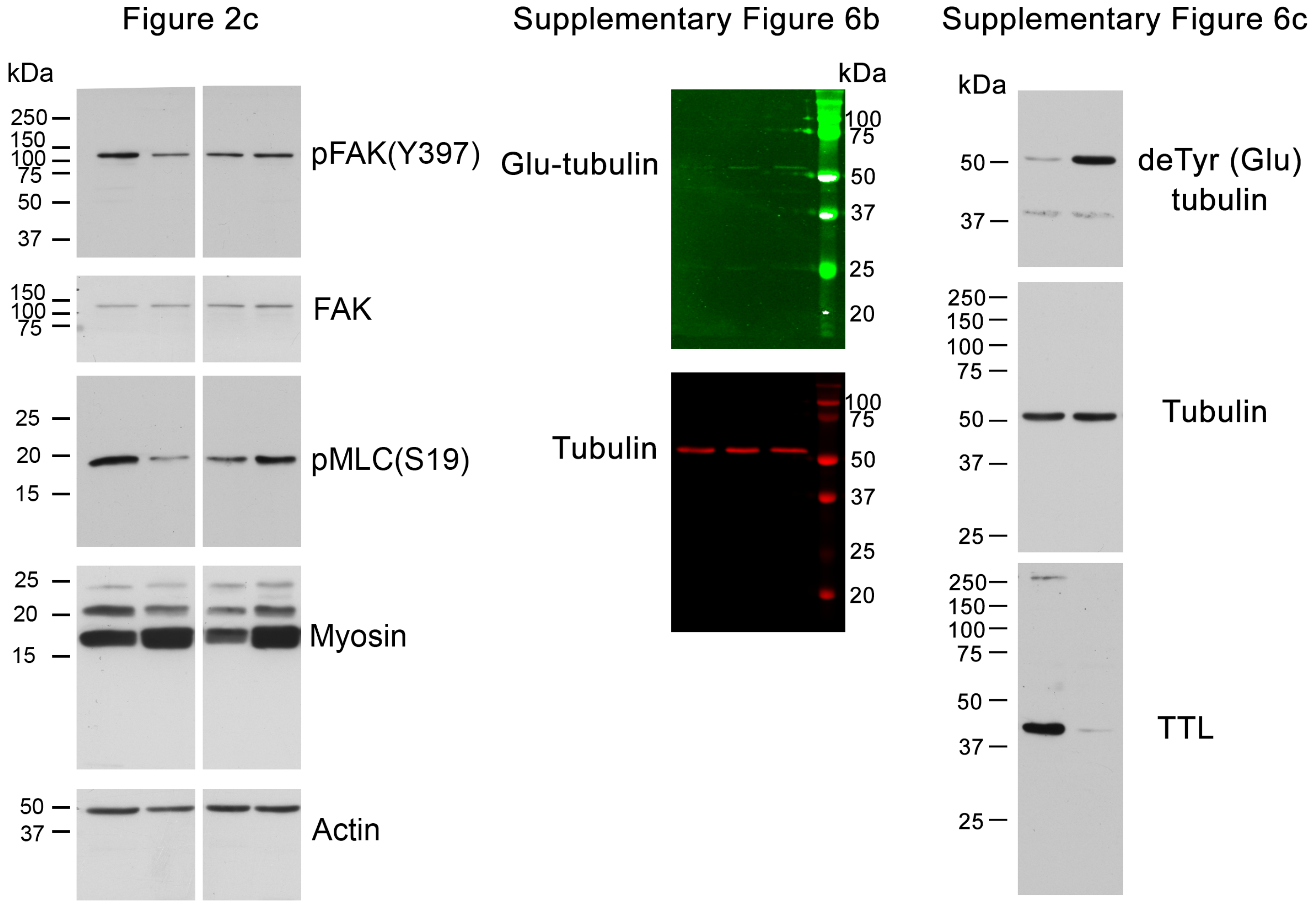

Supplementary Figure 10: Images of uncropped Western blots.
