# Supplementary material for: Extracellular matrix stiffness and cell contractility control RNA localization to promote cell migration
Source: Nat Commun. 2017 Oct 12;8:896. doi: 10.1038/s41467-017-00884-y (PMC5638855; doi:10.1038/s41467-017-00884-y)
Supplement: Supplementary file 3 — Description of Additional Supplementary Files [file 41467_2017_884_MOESM3_ESM.pdf]

Description of Additional Supplementary Files

File Name: Supplementary Data 1
Description: **RNA-Seq data of control and APC-knockdown cells**. Table lists all identified RNAs is protrusions and cell bodies together with associated fold changes and pvalues. Additional sheets list RNAs within specific groups and associated cutoffs.

File Name: Supplementary Data 2
Description: **Ingenuity Pathway Analysis (IPA) results**

File Name: Supplementary Data 3
Description: **RNA-Seq data of HBB and Pkp4 cUTR-expressing cells.** Table lists all identified RNAs is protrusions and cell bodies together with associated fold changes and pvalues. Additional sheets list RNAs within specific groups and associated cutoffs.
